# Effect of Polyimide-Phosphating Double Coating and Annealing on the Magnetic Properties of Fe-Si-Cr SMCs

**DOI:** 10.3390/ma15093350

**Published:** 2022-05-07

**Authors:** Haiming Long, Xiaojie Wu, Yunkun Lu, Haifeng Zhang, Junjie Hao

**Affiliations:** 1 Institute for Advanced Materials and Technology, University of Science and Technology Beijing, Beijing 100083, China; yingfengzui@163.com (H.L.); luyunkun123@126.com (Y.L.); 18503365756@163.com (H.Z.); 2 Avic Chengdu Caic Electronics Co., Ltd., Chengdu 610091, China; wuxiaojie2020@163.com

**Keywords:** Fe-Si-Cr SMCs, PI, core–shell double coating, annealing, magnetic properties

## Abstract

Fe-Si-Cr soft magnetic powder cores (SMCs), with high electrical resistivity, magnetic permeability, saturation magnetic induction, and good corrosion resistance, are widely applied to inductors, filters, choke coils, etc. However, with the development of electronic technology with high frequency and high power density, the relative decline in the magnetic properties limits the high-frequency application of SMCs. In this paper, the phosphating process and polyimide (PI) insulation coating is applied to Fe-Si-Cr SMCs to reduce the core loss, including hysteresis loss and eddy current loss. The microstructure and composition of Fe-Si-Cr powders were analyzed by SEM, XRD, and Fourier-transform infrared spectra, respectively. The structural characteristics of the Fe-Si-Cr @ phosphate layer @ PI layer core–shell double coating were studied, and the best process parameters were determined through experiments. For SMCs with 0.4 wt% content of PI, the relative permeability is greater than 68%, and the core loss is the lowest, 7086 mW/cm^3^; annealed at 500 °C, the relative permeability is greater than 57%, and the core loss is the lowest, 6222 mW/cm^3^. A 0.4 wt% content of PI, annealed at 500 °C, exhibits the ideal magnetic properties: μ_e_ = 47 H/m, *P* = 6222 mW/cm^3^.

## 1. Introduction

Metal magnetic powder cores, belonging to a kind of soft magnetic composite material, are prepared by mixing ferromagnetic powder with an insulating medium [1]. They are commonly used in transformers, electronic communication, and radar due to the strengths of high saturation induction density, high magnetic permeability, and low total loss [2,3]. In recent years, Fe-Si-Cr SMCs, as new soft magnetic composite materials, have widely been applied to the inductance, filter, choking ring, and similar areas due to their higher electronic resistivity, permeability, and lower core loss in comparison with traditional silicon steel sheets, single-metal-based soft magnetic materials [4]. In high-frequency applications, adding Si can not only reduce the eddy current loss by increasing the resistivity of SMCs but also can form CrSi and CrSi_2_ in Fe-Si-Cr SMCs with excellent temperature characteristics [5]. The addition of Cr can improve the mechanical strength, plasticity, and corrosion resistance of SMCs [6]. Compared with other iron-based SMCs, Fe-Si-Cr SMCs offer better broadband response characteristics and lower cost. Unfortunately, the increment in core loss as a result of increased operating frequency limits the large application of Fe-Si-Cr SMCs [7].

To reduce core loss *P_cv_*, including hysteresis loss *P_h_* and eddy current loss *P_e_*, insulating coating and high-pressure forming are usually applied in the manufacturing process [8,9,10,11,12,13,14,15,16,17,18]. Generally, there are two types of coatings used to suppress eddy currents: organic coatings and inorganic coatings [19]. With the advantages of satisfactory adhesion and flexibility, organic substances such as epoxy resin [20] or phenolic resin [21] have been used as the insulating layer of SMCs. Due to high dislocation density and defects, high pressure causes an increase in hysteresis loss *P_h_*. In order to eliminate defects such as lattice strain, a high-temperature annealing process is usually used. Some new characterization methods have promoted the study of SMCs [22,23,24]. However, the annealing process above 400 °C easily decomposes the organic resin [25]. Therefore, phosphate [26] and oxide [27,28] are used as the passivation layer of SMCs. However, the phosphate insulating layer will also collapse during the annealing process, resulting in a decrease in resistivity [26]. The organic coating has good adhesion but poor heat resistance. PI has higher heat resistance, insulation resistivity, and mechanical stability than ordinary resins, which is a potential organic coating material for magnetic powders. However, PI is a non-magnetic material; it can increase resistivity but decrease permeability [29]. To optimize the magnetic properties and reduce the core loss, high-temperature annealing is an effective method [30].

The process of preparing SMCs by powder metallurgy has been widely used to lower costs and improve efficiency [31,32]. This method is based on using each fine powder particle to make the insulating coating, which can significantly reduce the core loss of the SMCs. Therefore, the research on high-performance insulating coatings and coating methods for SMCs is currently a popular research subject [33]. Due to the excellent insulation performance of the organic coating but the decomposition temperature being low, it cannot be combined with subsequent high-temperature annealing treatment to eliminate the influence of residual stress during pressing. Therefore, the current research tends to use an inorganic coating to improve the annealing temperature [34]. There are few papers on the research of inorganic + organic double coating, especially research on using PI with high decomposition temperature as an organic coating. Although many inorganic materials also have excellent insulation properties and can significantly reduce eddy current loss, the compact density of inorganically coated SMCs is generally lower than that of those that are organically coated, so there will be defects, such as compact pores, which in turn affect magnetic properties, such as hysteresis loss.

In this paper, to reduce the core loss of SMCs at high frequencies, an inorganic phosphate + organic PI double coating with excellent insulation performance was used to improve powder resistivity and reduce eddy current loss; the organic coating PI with a higher decomposition temperature was used to increase the compact density and increase the annealing temperature to reduce the hysteresis loss. Finally, higher magnetic properties can be obtained by annealing in an argon atmosphere at 500 °C for 1 h.

## 2. Materials and Methods

### 2.1. Preparation of SMCs

**Figure 1:**
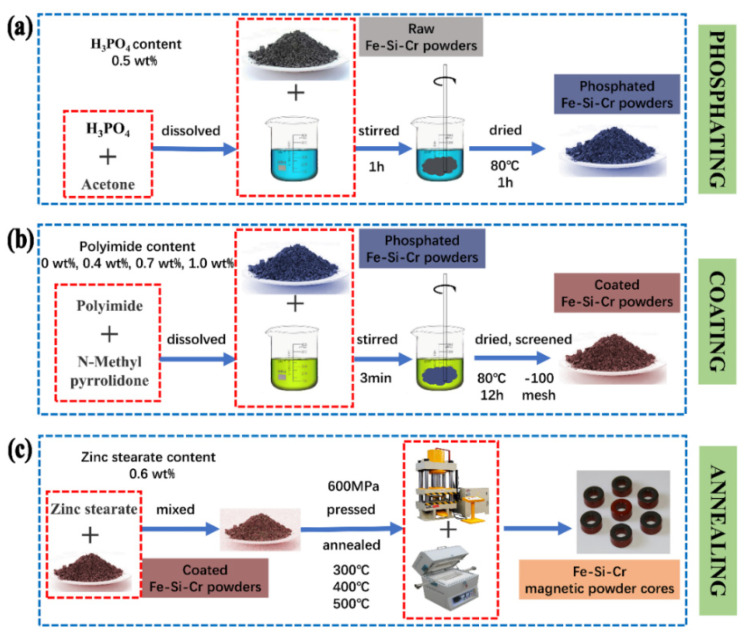
The preparation process of Fe-Si-Cr@PI core–shell structure coating and magnetic powder cores: (**a**) phosphating, (**b**) coating, and (**c**) annealing.

Fe-Si-Cr powders with d50 = 10 μm were prepared by gas atomization, consisting of 3.3 wt% Si, 6.5 wt% Cr, and balance of Fe. The preparation process of Fe-Si-Cr @ phosphate layer @ PI layer core–shell double coating and SMCs is divided into three steps: phosphating, coating, and annealing, as shown in Figure 1. First, Fe-Si-Cr powders were pretreated with phosphate, as shown in Figure 1a. The phosphating procedure was carried out in 50 mL of acetone, mechanical stirring at room temperature for one hour followed by drying at 80 °C for one hour. Secondly, we prepared PI coating, as shown in Figure 1b. The Fe-Si-Cr powders were mixed uniformly with the various PI of 0 wt%, 0.4 wt%, 0.7 wt%, 1.0 wt%, respectively (the PI cannot be dissolved into the water or alcohol solution and can be dissolved with *N*-Methyl pyrrolidone), and air-dried at 80 °C for 12 h. The dry powders were passed through a screen of −100 mesh. Finally, we prepared and annealed the SMCs; as shown in Figure 1c, the coated Fe-Cr-Si powder was uniformly mixed with zinc stearate lubricant (0.6 wt%). Next, the coated powders were pressed into cores under applied axial stress of 600 MPa with outer diameter of 14 mm, inner diameter of 8 mm, and height of about 3 mm. Lastly, the SMCs were annealed at different temperatures from 300 °C to 500 °C for one hour in argon atmosphere with a pipe furnace. 

### 2.2. Test Method and Material Characterization

**Figure 2:**
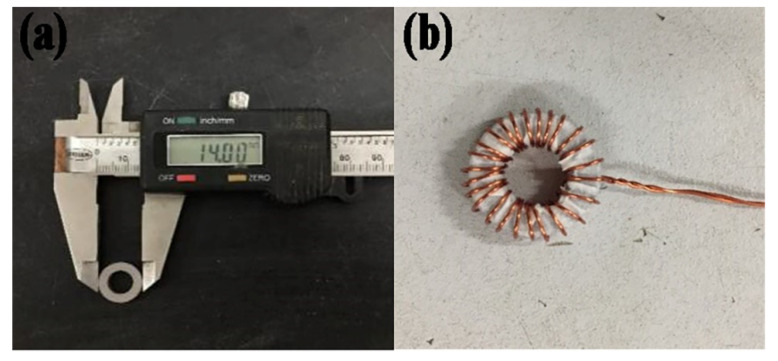
The SMCs sample to be tested: (**a**) magnetic powder core size and (**b**) coil winding before magnetic performance test.

The inductance of the Fe-Si-Cr SMCs was measured by the LCR bridge tester, and we calculated the magnetic permeability by using Equation (1):

(1) $$\mu_{e}=\frac{L\times 10^{-9}\times L_{e}}{4NA_{e}}$$

where *μ_e_* is the effective permeability, *L* is the inductance of sample core, and *L_e_* is the mean flux density path of the ring sample. *N* is the number of turns of the coil (*N* = 25), *A_e_* is the area of cross-section. Figure 2 shows the magnetic powder core to be tested.

The microstructure of uncoated and coated Fe-Si-Cr powder was characterized by scanning electron microscopy (SEM, LEO1450, CARL ZEISS, Oberkochen, Germany) equipped with the energy dispersive X-ray spectrometry (EDS, Quanta-200, CARL ZEISS, Oberkochen, Germany). FTIR was used to verify the phosphating effect and the coating effect of PI (Thermo Scientific Nicolet iS5, Thermo Fisher Scientific, Waltham, MA, USA). XRD was used to characterize the structure of the powder and SMCs (Rigaku Ultima IV, Rigaku Corporation, Tokyo, Japan). The kinetics of thermal decomposition of PI was investigated using synchronous thermal analyzer (TG-DSC, Q600, METTLER-TOLEDO, DE, USA). LCR bridge tester (TH2829C, Agitek, Xi‘an, China) is used to measure the inductance of SMCs, the core loss was measured by an auto testing system for SMCs (IWATSU SY-943, IWATSU ELECTRIC, Tokyo, Japan) in the frequency range of 100 kHz^−1^ MHz, and the magnetic flux density was set to 50 mT.

## 3. Results

### 3.1. Characteristics of Phosphated and Coated Layer

**Figure 3:**
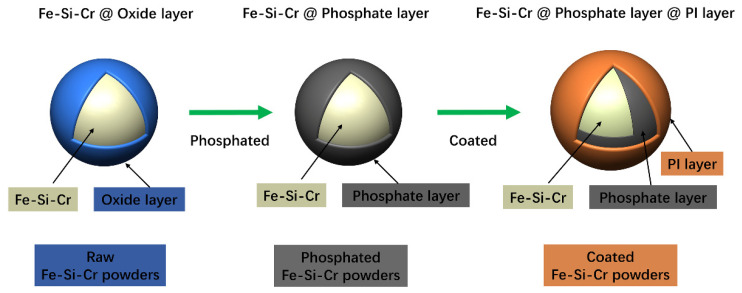
The effect of phosphating and coating process on the surface of Fe-Si-Cr powder particles.

After the two-step process of phosphating and coating, the oxide layer of the raw powder particles can be removed, and a certain thickness of the phosphate layer and PI insulation layer can be obtained, as shown in Figure 3. On the one hand, phosphating can remove the oxide layer on the surface of the original powder, including iron oxide, chromium oxide, and silicon oxide. On the other hand, a phosphating layer can be formed on the surface of powder in the phosphating process so as to increase the resistivity and reduce the eddy current loss [35]. PI is a non-magnetic material; it can increase resistivity but reduce permeability. To increase the insulation resistance without damaging the magnetic permeability, it is necessary to determine the appropriate content of PI addition—that is, to optimize the thickness of the Fe-Si-Cr @ phosphate layer @ PI layer core–shell double coating. 

#### 3.1.1. Characteristics of the Phosphated Layer

**Figure 4:**
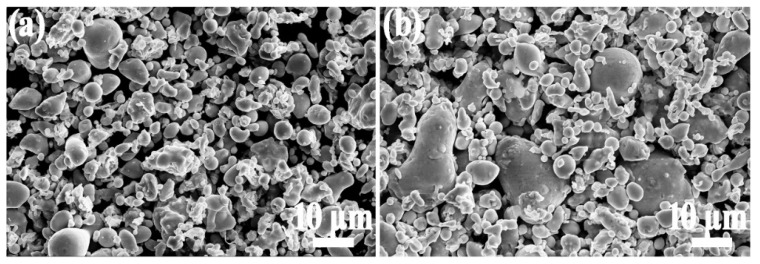
Comparison of Fe-Si-Cr powders: (**a**) Fe-Si-Cr raw powder, (**b**) Fe-Si-Cr phosphated powder.

The SEM images of the Fe-Si-Cr raw powder and the phosphated powder are shown in Figure 4. It can be seen from Figure 4a that the distribution of the Fe-Si-Cr raw powder particles is relatively dispersed, and most of the particles are spherical or nearly spherical (spindle shape). This is because the cooling rate of the gas atomization process is slower than that of the water atomization process, and it is easy to obtain a spherical powder. At the same time, for the surface oxide layer of powder particles, the gas-atomization process is much smaller than the water-atomization process. It can be seen from Figure 4b that the surface of the Fe-Si-Cr powder particles after phosphating is smooth, which indicates that the phosphate layer is evenly distributed on the surface of the powder. After the phosphating treatment, the phosphated substance—the reaction product of phosphoric acid, iron, and chromium—cannot be observed intuitively and is further characterized by other methods in a follow-up. 

**Figure 5:**
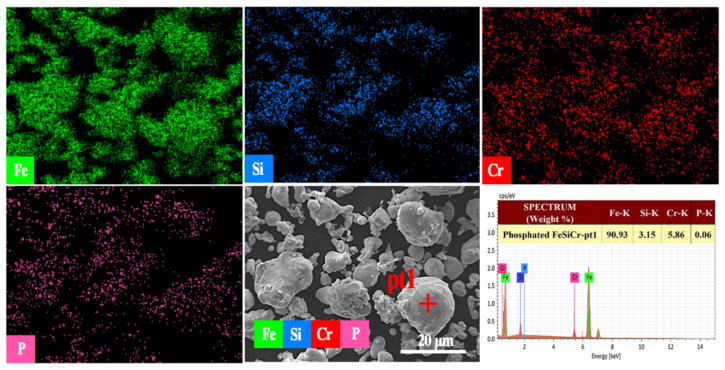
The EDS of Fe-Si-Cr phosphated powder.

The energy spectrum characteristics of the powder after phosphating are shown in Figure 5. It can be clearly observed that the *p* element is evenly distributed on the surface of the powder, which indicates that a phosphate layer is formed on the surface of the Fe-Si-Cr powder. The presence of phosphate can improve the resistivity of Fe-Si-Cr powder so as to ensure a relatively low eddy current loss and good processability [36].

#### 3.1.2. Characteristics of the PI Coating Layer

**Figure 6:**
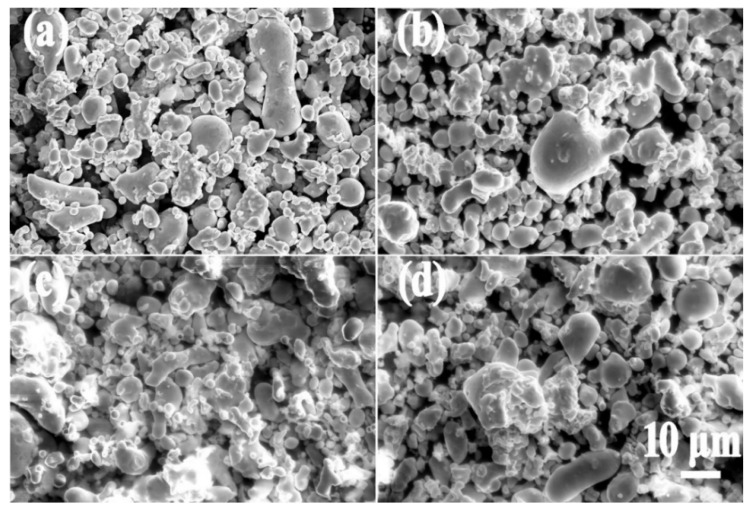
Fe-Si-Cr powders coated with PI: (**a**) 0 wt% PI, (**b**) 0.4 wt% PI, (**c**) 0.7 wt% PI, (**d**) 1.0 wt% PI.

Figure 6 is a microscopic image of Fe-Si-Cr powder coated with different content of PI. In Figure 6a, the Fe-Si-Cr powder is uncoated after phosphating. The powder is relatively dispersed and has an average particle size of 10 μm. In Figure 6b,c, the Fe-Si-Cr powder coated with PI is a mostly irregular, spherical powder. Meanwhile, there is a reunion as a result of the bonding effect of PI.

**Figure 7:**
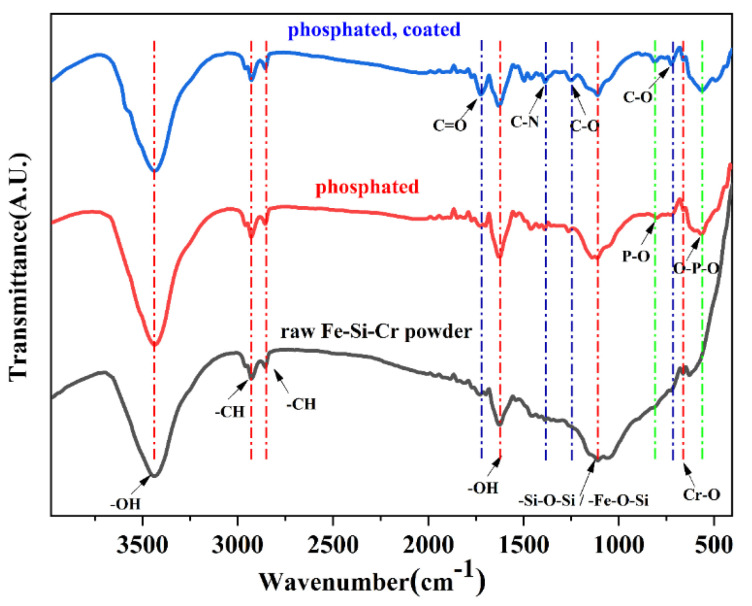
FTIR spectrum of uncoated and coated Fe-Si-Cr powder (from bottom to top is the raw powder, phosphated powder, and phosphate-coated powder.

Figure 7 shows the Fourier-transform infrared spectrum. It can be seen from the Fe-Si-Cr raw powder that the broad absorption peak at 3438 cm^−1^ is the -OH stretching vibration of adsorbed water, and the absorption peaks at 2928 cm^−1^ and 2855 cm^−1^ are the symmetric and asymmetric stretching vibrations of -CH in the methylene group. The absorption peak at 1626 cm^−1^ is the -OH bending vibration of water molecules, the absorption peak at 1110 cm^−1^ is the asymmetric stretching vibration of Si-O-Si or Fe-O-Si, and the absorption peak at 663 cm^−1^ is caused by the stretching vibration of Cr-O. For the phosphated powder, new absorption peaks appear at 567 cm^−1^ and 802 cm^−1^; the absorption peak at 567 cm^−1^ is the bending vibration of O-P-O and the asymmetric stretching vibration of P-O at 802 cm^−1^. According to these two absorptions, the presence of the peak can determine that the sample contains ${\mathrm{PO}}_{4}^{-3}$ and the intensity of the absorption peak at 1112 cm^−1^ becomes lower. It is possible that phosphoric acid interacts with Fe-O-Si, which reduces its content. For the phosphated and coated powder, new absorption peaks appeared at 1725 cm^−1^, 1387 cm^−1^, 1250 cm^−1^, and 724 cm^−1^. These absorption peaks are all caused by the characteristic peaks of PI. Among them, 1725 cm^−1^ is carbonyl C=O stretching vibration, 1387 cm^−1^ is C-N stretching vibration, 1250 cm^−1^ is C-O stretching vibration, and 720 cm^−1^ is C-O bending vibration, indicating that the powder is successfully coated with PI [37,38,39].

### 3.2. Effect of PI on the Phase Composition and Magnetic Properties

#### 3.2.1. Effect of PI Content on the Phase Composition of the Fe-Si-Cr SMCs 

**Figure 8:**
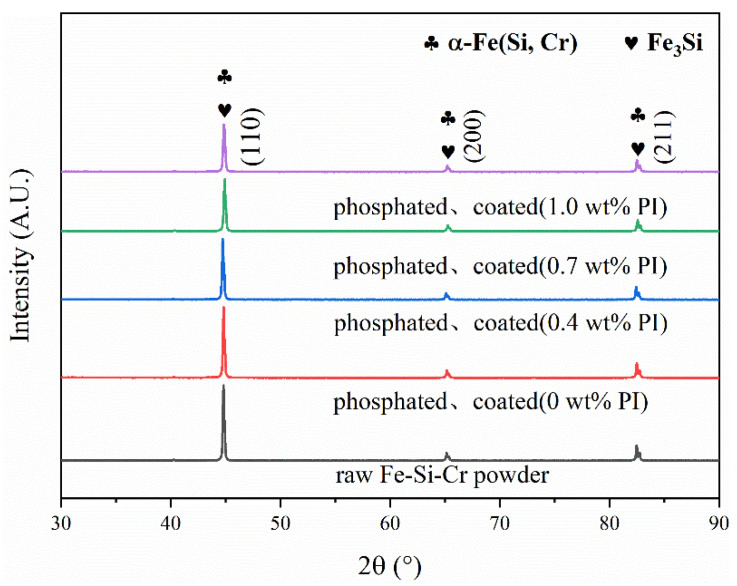
XRD patterns of Fe-Si-Cr powders with different PI coating contents.

Figure 8 is the XRD pattern of Fe-Si-Cr powders with different PI coating amounts. Three sharp characteristic peaks (110), (200), and (211) are detected, which are consistent with the peaks of the α-Fe and Fe_3_Si [40,41]. The Si and Cr atoms are solid-dissolved in the crystal lattice of α-Fe, and a solid solution of bcc -α-Fe (Si, Cr) is formed. It can be seen from the figure that the characteristic peak intensity after phosphating is significantly lower than the characteristic peak intensity before phosphating. This is due to the reaction of iron and phosphoric acid to form a phosphate layer, which reduces the characteristic peak intensity of α-Fe [42], and FTIR spectroscopy analysis also confirmed the existence of the phosphate layer. As the amount of PI coating increases from 0 to 1.0 wt%, the characteristic peak intensity also shows a downward trend. This is because the thickening of the PI layer weakens the X-ray absorption of the Fe-Si-Cr matrix. However, due to the thinner phosphate layer and PI layer, the XRD failed to detect the phosphide and PI phases.

#### 3.2.2. The Trend of Magnetic Properties with PI Content

**Figure 9:**
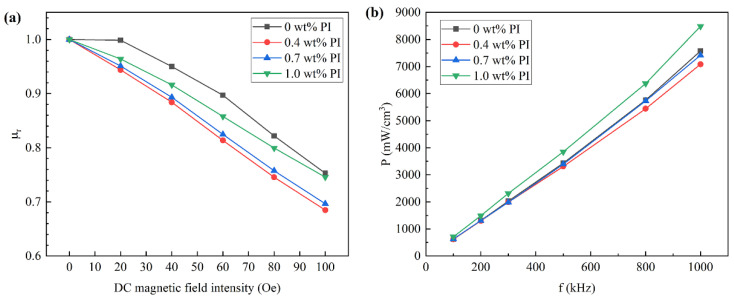
The trend of magnetic properties with PI content: (**a**) relative permeability; (**b**) core loss.

The magnetic properties of SMCs can be characterized by relative permeability and DC bias capability. DC bias refers to the superposition of an alternating current when an alternating magnetic field and DC magnetic field are simultaneously applied to the magnetic core. Figure 9a shows the DC bias capacity curve of Fe-Si-Cr SMCs coated with different contents of PI. It can be seen from the figure that the DC bias capacity of SMCs without PI is the best. When the applied magnetic field strength is 100 Oe, the relative permeability reaches 75%. Compared with the sample without PI, PI reduces the relative permeability of SMCs; however, when the magnetic field strength is 100 Oe and the PI content is 0.4 wt%, the relative permeability is >68%, indicating that its DC bias ability is not poor. In the range of 0~1.0 wt%, the relative permeability increases with the increase in PI content. This is because, in the applied DC magnetic field, SMCs are magnetized, the pressing density of SMCs without PI is low, and the air gap hinders the rotation and displacement of the magnetic domain, which makes it difficult for SMCs to be magnetized to saturation. However, the compaction density of SMCs with PI is relatively high, the air gap is small, and the displacement and rotation of the magnetic domain are relatively small, so it is easier to be magnetized to saturation. However, the addition of non-magnetic PI resin reduces the proportion of magnetic substances in SMCs. At this time, the resin hinders the rotation and displacement of magnetic domains, such as the gap between particles. Therefore, the relative permeability decreases compared with that without PI resin.

Figure 9b is the core loss curve of Fe-Si-Cr SMCs coated with different PI contents. The total loss (*P_cv_*) is composed of hysteresis loss (*P_h_*), eddy current loss (*P_e_*), and residual loss (*P_c_*). The residual loss is the micro-eddy current generated by the magnetic domain wall, which is very small compared to the hysteresis loss and eddy current loss. It can be ignored. The core loss of all the samples increases with the increase in frequency; at the same frequency, the SMCs have the smallest core loss at the 0.4 wt% PI. When the frequency is 1000 Hz, the core loss is 7086 mW/cm^3^. Due to the application of double-insulating coatings, the coated cores exhibit lower magnetic loss than uncoated cores; insulating coated layers effectively hinder the current of intra-particles and inter-particles and thus reduce the eddy current loss. When the PI content is 1.0 wt%, the core loss is the largest. Because the insulating layer is a non-magnetic substance, it acts as a hindrance in the magnetization process of the Fe-Si-Cr SMCs, resulting in an increase in hysteresis loss. When the insulating layer is thicker, the eddy current loss is reduced, while the hysteresis loss is increased so that the total core loss is increased.

### 3.3. The Effect of Annealing on the Phase Composition and Magnetic Properties

#### 3.3.1. The Choice of the Annealing Temperature and the Change of the Phase Composition

**Figure 10:**
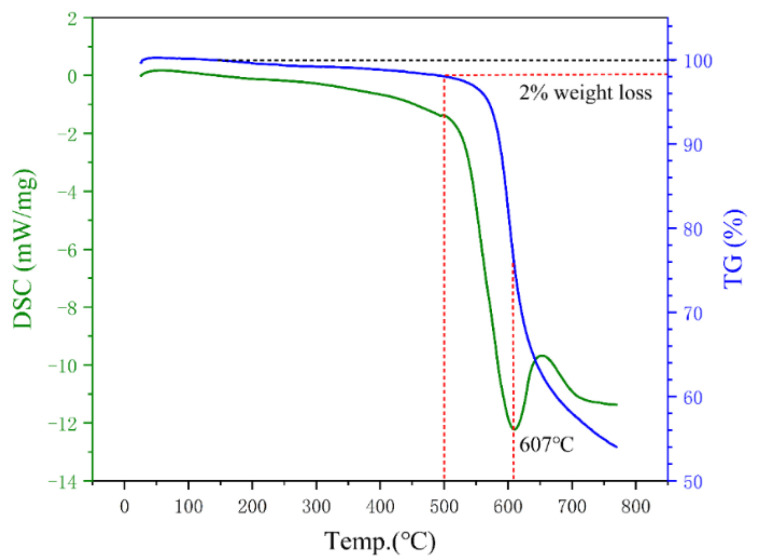
Kinetic curves of thermal decomposition of PI in argon.

From the point of view of eliminating residual stress, the higher the annealing temperature, the better the effect. However, the selection of the annealing temperature should consider the influence of the passivation layer and coated layer. Phosphating treatment will form a passivation layer on the surface of the powder, and the phosphating layer will crystallize with iron at 500 °C [43]. In addition, the final annealing temperature should be determined in combination with the heat resistance of the PI coating. As shown in Figure 10, according to the DSC curve, PI has an endothermic peak near 607 °C, which is the thermal decomposition temperature of PI. According to the TG curve, the 2% thermal weight loss temperature is as high as 500 °C (mainly due to the evaporation of adsorbed water in PI powder), and the maximum heat-resistant temperature of PI can reach 600 °C. Therefore, the final annealing temperature range is determined as 300~500 °C.

**Figure 11:**
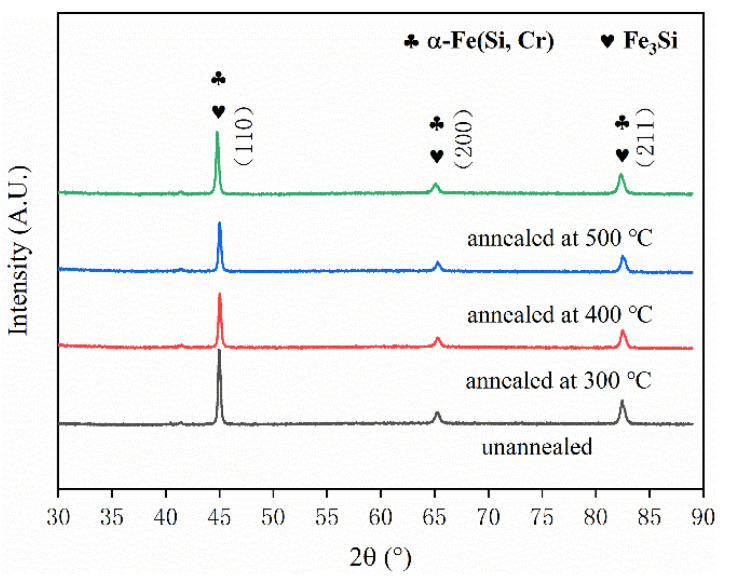
XRD patterns of Fe-Si-Cr SMCs at different annealing temperatures.

Figure 11 is the XRD pattern of Fe-Si-Cr SMCs with different annealing temperatures. Similar to Fe-Si-Cr powder, three sharp characteristic peaks (110), (200), and (211) are detected, and the phase composition is mainly α-Fe (Si, Cr) solid solution and Fe_3_Si. It can be seen that annealing only eliminates the internal stress of SMCs without changing their phase composition.

#### 3.3.2. The Trend of Magnetic Properties with Annealing Temperature

**Figure 12:**
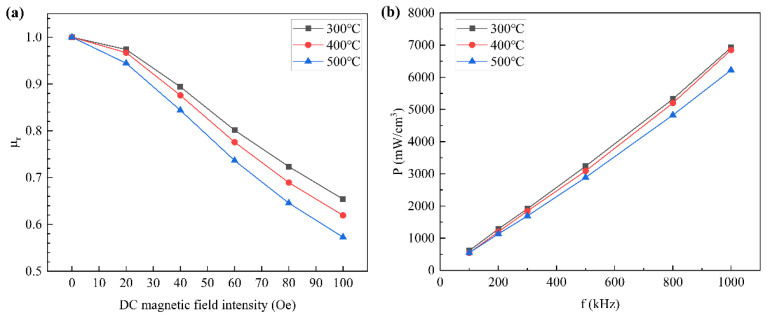
The trend of magnetic properties with annealing temperature (the powder was phosphated and coated with 0.4 wt% PI): (**a**) relative permeability; (**b**) core loss.

The pressing process will reduce the air gap and produce residual stress in the SMCs before the annealing process. Due to the reduction in non-magnetic materials and the increase in effective permeability, the DC bias ability becomes worse. The trend of DC bias capacity with heat treatment is shown in Figure 12a; the relative permeability of the SMCs annealed at 300 °C is the highest, reaching 69% at 100 Oe. The relative permeability of cores reduced gradually with the decrease in the annealing temperature from 300 °C to 500 °C. This is because the higher the annealing temperature, the lower the domain wall resistance, and the corresponding magnetic core is easily magnetized to saturation. However, under a 100 Oe magnetic field intensity, the magnetic permeability of the SMCs annealed at 500 °C reaches 57%, which also does not show a poor DC bias.

Annealing can eliminate the residual internal stress and dislocation generated after magnetic particle pressing, compact the structure, reduce air and other defects, reduce the hysteresis loss coefficient, and finally, reduce the hysteresis loss. The higher the annealing temperature, the more thorough the removal of internal stress, air and dislocation, and other defects between magnetic particles, and the more obvious the effect of loss reduction [44,45]. Figure 12b shows the core loss of Fe-Si-Cr SMCs annealed at different temperatures. It can be seen from the figure that the iron loss decreases gradually with the increase in annealing temperature. The core annealed at 500 °C has the lowest loss, which is only 6222 mW/cm^3^ at 1000 Hz.

### 3.4. The Effect of PI Content and Annealing Temperature on Effective Permeability

**Figure 13:**
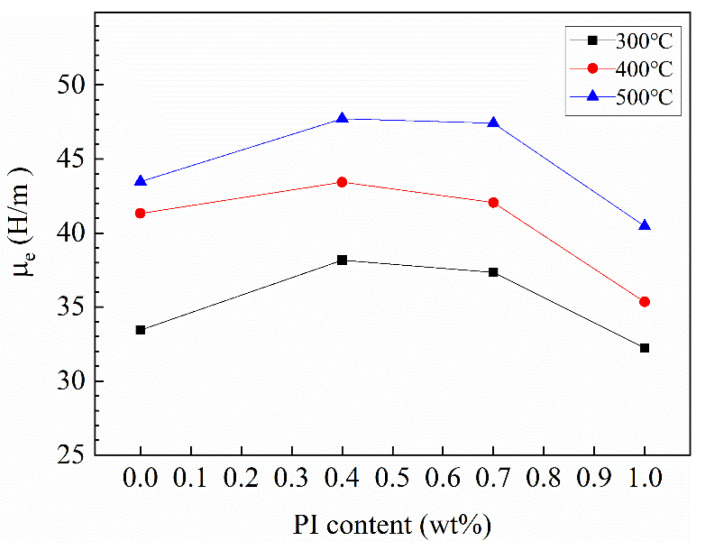
Trend of PI content and annealing temperature on effective permeability of Fe-Si-Cr SMCs.

Figure 13 shows the effective permeability of Fe-Si-Cr SMCs at different PI contents and different annealing temperatures. It can be seen that all the samples show the same trend; with the increase in PI content, the effective permeability increases first and then decreases at the same temperature. The phenomenon can be ascribed that the increase in PI leads to the high compaction density of the powder, and thereby the air gap decreases and the effective permeability increases, which can be confirmed in Table 1. The density of core coated at 0.4 wt% PI and annealed at 500 °C reached 6.213 g/cm^3^. However, PI is a non-magnetic substance, and the increase in PI causes a decrease in magnetic material and a decrease in effective permeability. The best response was obtained for the sample coated with 0.4 wt% PI. During annealing, the atomic disorder state was changed to an ordered state, the microstructure of Fe-Si-Cr SMCs was optimized well, the air gap was reduced, and the annealed cores were denser, and it can be seen in Table 1 that the density of cores increased with the increase in annealing temperature. Thus, improving the effective permeability of Fe-Si-Cr SMCs, the SMCs show an ideal effect at an annealing temperature of 500 °C. The Fe-Si-Cr SMCs, with 0.4 wt% content of PI and heat treatment temperature at 500 °C, exhibited the best magnetic properties: *μ_e_* = 47 H/m, *p* = 6222 mW/cm^3^.

In the field of electromagnetism, the total core loss (*P_cv_*) consists of hysteresis loss (*P_h_*), eddy current loss (*P_e_*), and residual loss (*P_c_*); the residual loss is the micro-eddy current generated by the domain wall, which is very small compared with the hysteresis loss and eddy current loss and can be ignored. Additionally, the total core loss *P_cv_* can be expressed as Equation (2) [14,17].

(2) $$P_{cv}=P_{h}+P_{e}=K_{h}\times f+k_{e}\times f^{2}$$

where *K_h_* is the hysteresis loss coefficient, *K_e_* is the eddy current loss coefficient, and *f* is the frequency. At low frequencies, the increase in total loss is mainly the increase in hysteresis loss, while at medium and high frequencies, the increase in total loss is mainly eddy current loss. The comparison of magnetic properties between this study and the literature is shown in Table 2. In this study, the effects of inorganic + organic double coating and heat treatment on the total core loss of SMCs are preliminarily explored; however, more accurate quantitative research on hysteresis loss and eddy current loss has not been completed. In further research, the quantitative results of the influence on each component of core loss *P_cv_* will be emphatically considered, and the effects of different process steps, including powder coating preparation, pressing, and annealing on hysteresis loss *P_h_* and eddy current loss *P_e_* will be evaluated so as to provide guidance for industrial production. In addition, the use of organic PI coating can significantly improve the corrosion resistance of SMCs, which is also worthy of further research.

## 4. Conclusions

In this paper, to reduce the core loss of SMCs in high-frequency application environments, the strategies of inorganic–organic double-insulating coating and high-temperature annealing were adopted. Phosphating can not only remove the oxide layer on the powder surface but also form a phosphate insulating coating on the powder surface. This insulating coating can significantly reduce the core loss of SMCs. At the same time, this phosphate is also a good intermediate transition layer for coating organic PI; SMCs coated with organic PI can significantly reduce the core loss, the addition of PI can increase the lubricity of powder in the pressing stage of SMCs, cause the pressed compact have high density, and reduce the existence of defects such as pores, which is conducive to reducing the core loss. Annealing is an effective method to reduce the influence of the pressing process on the magnetic properties of SMCs, which can significantly reduce the core loss. In this study, the Fe-Si-Cr SMCs, with 0.4 wt% content of PI and annealing temperature at 500 °C, exhibit the best magnetic properties: *μ_e_* = 47 H/m, *p* = 6222 mW/cm^3^.

## Figures and Tables

**Table 1:** Density tests of Fe-Si-Cr SMCs coated with various content of PI at different annealing temperatures.

ρ (g/cm^3^)		FeCrSi/PI Content (wt%) in This Study				FeCrSi/Sodium Silicate [40]
		0	0.4	0.7	1.0	
Temperature (°C)	300	5.917	6.130	6.066	5.869	/
	400	6.051	6.206	6.146	5.920	/
	450	/	/	/	/	5.610
	500	6.163	6.213	6.179	6.112	/
	550	/	/	/	/	6.020
	650	/	/	/	/	6.140

**Table 2:** Comparison of the magnetic performances in this study and the literature.

Samples	*μ_e_* (H/m)	*P_cv_* (mW/cm^3^)			
		0.02T 1000 kHz	0.05T 100 kHz	0.05T 500 kHz	0.05T 1000 kHz
In this study	47.5	/	547	2888	6222
FeSiCr/phosphate [36]	44.5	/	780	/	/
FeSiCr/PA6 [42]	18	/	1100	/	/
FeSiCr/MnZnFe [18]	55	/	738	/	/
FeSiCr/yttrium nitrate [32]	41	600	/	/	6250
FeSiCr/carbonyl iron [34]	37	560	/	/	/
FeCrSi/sodium silicate [40]	34.9	/	/	/	/

## Data Availability

Not applicable.
